# Costs of Academic Engagement in Organized Plastic and Reconstructive Surgery in the United States

**DOI:** 10.1055/a-2832-6764

**Published:** 2026-05-29

**Authors:** Kishan S. Shah, Stefan T. Prvulovic, David H. Song

**Affiliations:** 1 Departments of Plastic and Reconstructive Surgery, Georgetown University School of Medicine, Washington, District of Columbia, United States; 2 Department of Plastic and Reconstructive Surgery, MedStar Georgetown University Hospital, Washington, District of Columbia, United States

**Keywords:** academic engagement, plastic and reconstructive surgery, financial barriers, cost burden, professional development, conference, society

## Abstract

Academic engagement in plastic and reconstructive surgery (PRS) has grown substantially, marked by an increasing number of professional societies, annual meetings, and peer-reviewed journals. While this growth has facilitated innovation, collaboration, and knowledge sharing, it has also introduced financial burdens. These costs may limit access to academic involvement, especially for early-career surgeons and those without institutional funding. Despite the implications these financial barriers pose for equity, academic engagement, and innovation within the field, the cumulative cost of academic involvement in PRS remains poorly quantified. A cross-sectional review was conducted to assess the financial costs of academic engagement in PRS in the United States. Between January and April 2025, data were collected from official websites of national and subspecialty PRS societies, journals, continuing medical education (CME) platforms, and board-certifying bodies. Annual dues, meeting fees, CME costs, journal subscription, publishing fees, and board-related expenses were compiled. A comparative analysis with five other surgical subspecialties was conducted. Thirty-two major PRS societies were identified. Membership dues ranged from $150 to $1,299. Meeting registration was $250 to $1,495. Journal subscription costs ranged from $44.90 to $1,518, and open-access (OA) article processing charges (APCs) from $700 to $5,334. PRS-board certification costs averaged $9,045. CME cost-per-credit reached up to $600. Among the six surgical specialties in the United States that were included in the comparative analysis, PRS ranked second in baseline academic engagement (BAE) costs after Neurosurgery, averaging $10,109. Academic engagement in PRS carries significant financial burdens that may limit access. Addressing these barriers is essential to maintaining equity and innovation.

## Introduction

Plastic and reconstructive surgery (PRS) has traditionally been defined by its blend of artistic vision, scientific innovation, and creative interdisciplinary collaboration. The establishment of professional academic bodies in the United States, such as the American Association of Plastic Surgeons (AAPS) in 1921 and the American Society of Plastic Surgeons (ASPS) in 1931, was foundational to the formal organization and advancement of PRS. 1 2 Born out of the need to address devastating wartime injuries during the World War I era, AAPS is the oldest PRS society and remains academically focused, supporting young surgeon-scientists through initiatives like the Academic Scholarship Program and the Constable International Travelling Fellowship. 2 The ASPS subsequently emerged as a central and unifying organization aimed at advancing PRS through national and global efforts in research, education, and professional development.

**Fig. 1:**
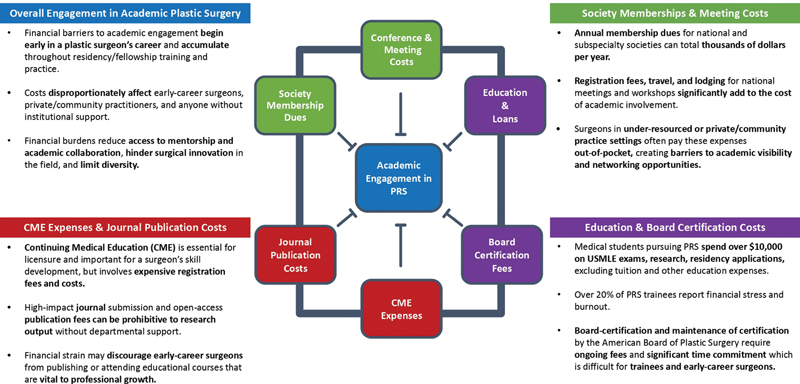
This figure showcases the six key components necessary to successfully engage in academic PRS. These components can be grouped into three subcategories, including CME expenses/journal publication costs, society membership/conference costs, and education/board-certification costs. The monetary investment required to achieve each component poses a significant financial burden to the trainee/surgeon and, ultimately, hinders academic engagement within the field. PRS, plastic and reconstructive surgery; USMLE, United States Medical Licensing Exam.

More recently, other well-respected organizations have emerged, such as the Plastic Surgery Research Council (PSRC), which is focused on cutting-edge translational PRS research, and The Aesthetic Society (TAS), which is centered on advancing aesthetic surgery research, techniques, and technologies. The success of these newer societies emphasizes PRS's expanding breadth and diversity. 3 Scholarly PRS journals, such as *Plastic and Reconstructive Surgery* , have also been key to the field's academic advancement, serving as hubs for capturing landmark innovations and promoting knowledge dissemination. 4 5 However, this unprecedented growth within the field has raised several concerns about the financial burden and the accessibility of sustained academic involvement in PRS, especially for trainees, early-career surgeons, and those without institutional support ( Fig. 1 ). 6 7 Costs such as society dues, board certification, conference fees, and journal submission present significant barriers to remaining academically engaged. 8 9

**Fig. 2:**
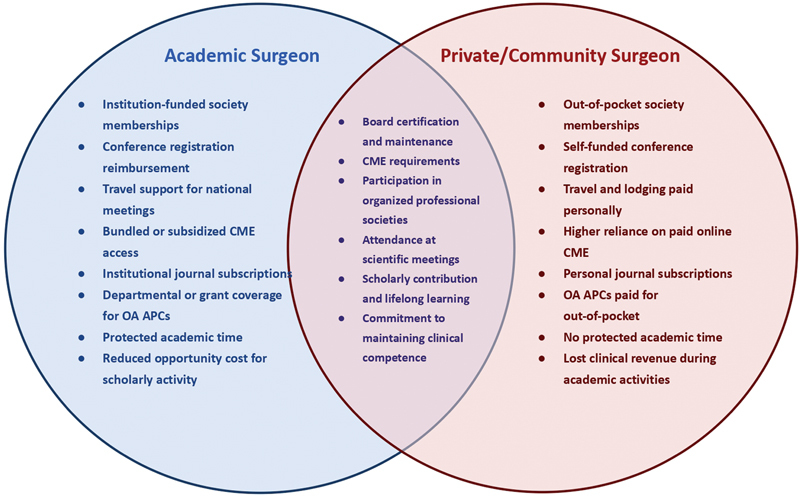
This conceptual figure illustrates differences across key domains of academic engagement between academic plastic surgeons and those in private or community practice. Domains include society memberships, conference attendance, CME credits, scholarly activity (Journals/OA APCs), and opportunity cost. This diagram is not intended to represent differences in overall compensation or net financial burden, as academic and private practice/community-based practice pathways involve distinct compensation models and professional trade-offs. This figure is intended solely to contextualize variations in support mechanisms for academic participation. APC, article processing charge; CME, continuing medical education; OA, open access.

The costs associated with academic PRS begin early. Medical students and residents face several expenses, including the United States Medical Licensing Exam (USMLE) fees, research/conference costs, residency applications/interview fees, and board-certification fees, which can exceed $10,000, especially in competitive specialties. 10 11 12 These costs are compounded by medical education debt, which contributes to burnout and dissatisfaction. Recent research shows that more than 20% of PRS trainees have educational debt exceeding $300,000. 13 14 After completing training, attending plastic surgeons continue to face ongoing expenses for maintaining the American Board of Plastic Surgery (ABPS) certification, journal submissions, society memberships, and conferences. While some academic surgeons receive institutional support, many surgeons in community and private practice settings must “pay out of pocket” for these costs ( Fig. 2 ). 8 15

These financial burdens have broader implications. They may deter individuals from lower socioeconomic backgrounds from pursuing academic PRS, reducing diversity within the field. 16 High costs associated with professional societies and conferences can also limit access to continuing medical education (CME), collaboration, and career advancement, which may lower the quality of care and hinder innovation. 9 17 The long-term sustainability of academic engagement in PRS requires urgent attention and support.

There is a paucity of literature that comprehensively analyzes the cumulative and specific costs incurred at each stage of academic engagement in the career path of a plastic surgeon in the United States. Therefore, the primary objectives of this study were to systematically identify and evaluate the financial costs and burden of becoming and remaining an academically active plastic surgeon in the United States using a data-driven approach, and to create greater transparency and stimulate discussion around the equity and accessibility of academic engagement in PRS.

## Methods

Data for this cross-sectional review study were collected between January and April 2025 through a systematic search of the official websites of national, regional, and subspecialty PRS societies, academic journals, CME websites, and board-certifying organizations.

This study focused on major PRS societies through a consensus author selection process in an effort to be as comprehensive as possible of national societies and known subspecialty societies ( Table 1 ). For each society, annual membership dues (stratified by medical student, resident/fellow, and attending/practicing surgeon status), registration fees for the annual meeting, and publicly posted costs for CME courses or sponsored educational programs were recorded. Organizations were contacted directly via email or phone if publicly available information, including pricing structures, unlisted membership categories, or missing organization details (e.g., year founded or total membership size), was incomplete or unclear. The costs reported in this study reflect standard publicly listed fees for the 2024–2025 cycle and exclude promotional discounts, early-bird registration rates, institutional subsidies, or negotiated pricing.

**Table 1:** Inclusion criteria for plastic and reconstructive surgery societies

Inclusion criteria	Description
**Society headquarters**	Society is U.S.-based or has a U.S. headquarters. Societies with a strong international presence were still included if the administrative center was in the United States
**Membership eligibility**	Society offers membership predominantly to plastic and reconstructive surgeons.
**Focus area**	Society's initiatives focus on PRS in general or a specific subspecialty within PRS.
**Establishment status**	Society is reasonably well-established with strong membership numbers, regular events/meetings, CME opportunities, and resources for surgeons.
**Annual events**	Society plans and hosts at least one annual event/conference/meeting.

Abbreviations: CME, continuing medical education; PRS, plastic and reconstructive surgery.

PRS journals were included based on reputation and impact factor, and information on annual individual subscription prices, submission fees, and open-access (OA) article processing charges (APCs). When applicable, distinctions were made between online-only versus print subscription, tiered pricing structures, affiliations with societies, and OA versus hybrid publishing models. Cost-per-credit ratios for CME were calculated for both traditional (e.g., in-person society meetings) and non-traditional (e.g., online modules) educational pathways.

To quantify the cost of becoming and remaining board-certified in PRS in the United States, the latest USMLE registration fees and all ABPS certification expenses and fees were recorded. 18 19 These costs were used to determine cumulative financial costs for PRS surgeons who desire ongoing active academic involvement in PRS. Annual ABPS recertification costs were excluded from the estimated “Total Cost” amount but were recorded for completeness.

A comparative analysis was conducted using available data on other surgical specialties to place the cost burden of academic participation in PRS into perspective. This comparison was based on the “baseline academic engagement” (BAE) model. Originally developed by Covell et al, BAE is defined as the cost of maintaining two annual society memberships, attending two annual meetings, and completing a one-time board certification. 20 Using these methods, the BAE cost was calculated for comparable specialties to PRS. This metric allowed for meaningful comparisons between specialty costs using up-to-date data. All costs were recorded in U.S. dollars and represent standard fees for 2024–2025. For the purposes of the BAE model only, board certification costs were defined as standardized, mandatory application and examination fees required for initial certification (written and oral examinations). Since board certification fee structures differ greatly across specialties, particularly with regard to the labeling and bundling of application, certification, and examination fees, we aimed to standardize inclusion criteria by capturing the mandatory application or certification fee (as defined by each respective board) and all required examination fees (written and/or oral) necessary for initial certification. Universal examinations (e.g., USMLE), longitudinal maintenance fees, recertification fees, and other administrative fees were excluded to preserve cross-specialty comparability. Selection criteria for societies for the BAE model prioritized broad organizational scope, high levels of engagement among practicing surgeons in the United States, and relevance to both academic and clinical practice, rather than global membership size alone. Annual meetings associated with these societies were chosen for consistency. Promotional discounts, early-bird rates, and institution-specific subsidies were not accounted for.

## Results

### Major Societies in Plastic and Reconstructive Surgery and Associated Fees/Costs/Dues

About 32 major PRS societies in the United States were identified ( Table 2 ). Of the 32 major PRS societies included, direct contact via email or phone was required for 23 societies. The three largest societies, by membership, were the ASPS (>11,000), the International Society of Aesthetic Plastic Surgery (ISAPS; >5,000), and TAS (>4,000). Membership dues varied widely between different societies, due to several factors, such as society size by membership, number of meetings or events held per year, and resources available for members. Annual dues for practicing plastic surgeons ranged from $150.00 (Migraine Surgery Society [MSS] and International Society of Craniofacial Surgery [ISCS]) to $1,299 (ASPS). Many societies offered special memberships for resident physicians (residents) and physicians in an accredited fellowship program (fellows) at a reduced cost, ranging from $25.00 (MSS and Virginia Society of Plastic Surgeons [VASPS]) to $425.00 (American Society for Surgery of the Hand). While many societies offered medical student membership for free, some societies offered medical student membership at a nominal cost, ranging from $30.00 (American Cleft Palate-Craniofacial Association [ACPCA]) to $200.00 (Southeastern Society of Plastic and Reconstructive Surgeons [SESPRS]).

**Table 2:** Membership costs of major societies in plastic surgery, organized by size and subspecialty

	Annual cost ($)				Other information	
Society/Organization name	Initiation/Application fee ($)	Practicing plastic surgeon	Resident/Fellow	Medical student	Total members	Year of founding
**National**						
American Society of Plastic Surgeons (ASPS)	150	1,299	100	50	11,000+	1931
American Association of Plastic Surgeons (AAPS)	0	635	N/A	N/A	600+	1921
Plastic Surgery Research Council (PSRC)	0	275	50	50	1,000+	1955
**Regional/State**						
California Society of Plastic Surgeons (CSPS)	50	500	75	0	450+	1951
Southeastern Society of Plastic and Reconstructive Surgeons (SESPRS)	25	650	0	200	650+	1958
New York Regional Society of Plastic Surgeons (NYRSPS)	a	a	a	a	300+	1960
Florida Society of Plastic Surgeons (FSPS)	100	850	N/A	N/A	150+	1956
Texas Society of Plastic Surgeons (TSPS)	0	750	100	50	360+	1951
Virginia Society of Plastic Surgeons (VASPS)	0	300	25	N/A	75+	1955
Northwest Society of Plastic Surgeons (NWSPS)	0	200	N/A	N/A	350+	1946
New England Society of Plastic Surgeons (NESPS)	25	225	0	N/A	250+	1931
New England Society of Plastic and Reconstructive Surgeons (NESPRS)	0	300	0	N/A	150+	1931
Ohio Valley Society of Plastic Surgeons (OVSPS)	0	200	N/A	N/A	60+	1958
Robert H. Ivy Pennsylvania Society of Plastic Surgeons (RHIS)	0	300	0	0	50+	1944
Minnesota Society of Plastic Surgeons (MSPS)	0	175	N/A	N/A	80+	1965
Mountain West Society of Plastic Surgeons (MWSPS)	0	100/150 b	0	N/A	150+	1955
**Aesthetic surgery**						
The Aesthetic Society (TAS) c	250	1,475	0	0	4,000+	1967
International Society of Aesthetic Plastic Surgery (ISAPS)	N/A	350	0	N/A	5,000+	1970
American Academy of Cosmetic Surgery (AACS)	100	1,000	0	N/A	8,000+	1985
**Breast surgery**						
American Society of Breast Surgeons (ASBrS)	0	595	145	25	3,800+	1995
**Hand surgery**						
American Society for Surgery of the Hand (ASSH)	700	750	420	N/A	3,900+	1946
American Association for Hand Surgery (AAHS)	75	500	0	N/A	1,500+	1981
**Advanced wound/burn**						
Wound Healing Society (WHS)	0	225	N/A	N/A	2,200+	1985
American Burn Association (ABA)	0	475	80	80	2,500+	1967
**Reconstructive/Microsurgery**						
American Society for Reconstructive Microsurgery (ASRM)	0	450	0	N/A	850+	1982
American Society for Peripheral Nerve (ASPN)	200	250	0	N/A	300+	1990
**Craniofacial**						
American Society of Craniofacial Surgeons (ASCFS)	0	200	0	N/A	400+	1983
American Society of Maxillofacial Surgeons (ASMS)	50	400	0	N/A	500+	1947
International Society of Craniofacial Surgery (ISCFS)	75	150	75	N/A	300+	1983
American Cleft Palate-Craniofacial Association (ACPA)	50	295	150	30	2200+	1943
Migraine Surgery Society (MSS)	0	150	25	0	80+	2017
**Gender affirming surgery**						
World Professional Association for Transgender Health (WPATH)	0	225	35	35	2,000+	1979

N/A denotes that there is no membership/pricing option for that particular type of attendee/member.

Although the American Academy of Cosmetic Surgery (AACS) was included for completeness, it was not taken into account for manuscript analyses, since many members are non-American Board of Plastic Surgery (ABPS)-board-certified plastic and reconstructive surgery surgeons.

a NYRSPS dues are now combined with ASPS dues.

b In-region pricing/out-of-region pricing.

c Formerly known as ASAPS Annual Meeting.

### Annual Meeting/Conference Costs

About 34 major PRS annual conferences and meetings in the United States were identified ( Table 3 ). Costs of attending annual PRS meetings were categorized by membership status and level of training. Practicing plastic surgeons with a membership to a particular society paid conference registration fees ranging from $250.00/300.00 (MSS/Robert H. Ivy Pennsylvania Plastic Surgery Society) to $1,495.00 (Florida Society of Plastic Surgery's [FSPS]Annual Meeting). Non-member rates were higher than member rates, and almost all societies offered discounted registration fees for resident physicians, fellows, and medical students, regardless of membership status.

**Table 3:** Costs of attendance at major plastic surgery annual meetings/conferences based on role and membership status

	Cost ($)					
Annual meeting name	Plastic surgeon member	Resident/Fellow member	Medical student member	Plastic surgeon non-member	Resident/Fellow non-member	Medical student non-member
**National**						
ASPS Annual Meeting - Plastic Surgery The Meeting (PSTM)	1,450	535	535	2,115	900	900
AAPS Annual Meeting	1,050	N/A	N/A	1,250	600	600
PSRC Annual Meeting	800	525	525	825	525	525
**Regional/State**						
CSPS Annual Meeting	600	225	225	1,200	225	225
SESPRS Annual Scientific Meeting	1,180	200	N/A	1,780	200	700
NYRSPS Annual Meeting	0	0	0	50	50	50
FSPS Annual Meeting	1,495	N/A	N/A	1,700	450 a	450
TSPS Annual Meeting	750	100	50	825	100	50
VASPS Annual Meeting	975	150	N/A	1,175	150	150
NWSPS Annual Meeting	750	N/A	N/A	1,050	450	450
NESPS Annual Meeting	650	N/A	N/A	750	100	100
NESPRS Annual Meeting	825	290	N/A	925	290	290
OVSPS Annual Meeting	700	N/A	N/A	700+	200+	150+
RHIS Annual Meeting	300	150	150	500	N/A b	N/A b
MSPS Annual Meeting	0	N/A	N/A	N/A	N/A	N/A
MWSPS Annual Meeting	600	300	N/A	800	325	325
**Aesthetic surgery**						
The Aesthetic Society (TAS) Annual Meeting - The Aesthetic MEET	1,275	100	100	1,875	100	100
ISAPS Annual Meeting	650	350	N/A	1,000	600	N/A
AACS Annual Meeting	1,399	399	0	1,699	499	0
Baker Gordon Annual Educational Symposium (The Aesthetic Society, ISAPS, and ASPS)	1,800	1,000	N/A	1,850	1,000	N/A
**Breast surgery**						
ASBrS Annual Meeting	795	400	400	1,395	1,395	1,395
**Hand surgery**						
ASSH Annual Meeting	1,045	1,045	1,045 c	1,255	1,255	1,255 c
AAHS Annual Meeting	925	N/A	N/A	1,325	325	325
**Limb/Extremity/Reconstructive surgery**						
Diabetic Limb Salvage (DLS) Annual Meeting	799	150	N/A	899	175	0
**Advanced wound/burn**						
WHS Annual Meeting	639	479	N/A	639	479	N/A
ABA Annual Meeting	999	999	349	1,099	1,099	449
**Microsurgery**						
ASRM Annual Meeting	950	800	N/A	1400	700	500
ASPN Annual Meeting	500	0	0	600	0	0
**Craniofacial**						
ASCFS/ACPA Combined Annual Meeting	650	75	75	965	275	275
ASMS Annual Meeting d	d	d	d	d	d	d
ISCFS Annual Meeting	1,300	550	N/A	1250	700	700
MSS Symposium e	275	50	50	375	150	150
**Gender affirming surgery**						
WPATH Annual Meeting	755	755	440	920	920	920
**Identity groups**						
Woman Plastic Surgery Symposium (ASPS Sponsored)	800	400	N/A	N/A	N/A	N/A

N/A denotes that there is no membership/pricing option for that particular type of attendee/member.

a Only for residents who are out of state. Residents from Florida can register free of charge.

b In order for fellows, residents, and medical students to attend the annual RHIS conference, they must be members of the RHIS.

c Prices may vary if a medical student qualifies for a guest pass or not.

d ASMS' annual meeting is held during a single day during the annual PSTM meeting. Participants must register for PSTM.

e The Migraine Surgery Symposium is held during the annual PSTM meeting.

### Journal Subscription and Open-Access Article Processing Costs

Journal subscription costs varied based on several factors, such as reputability and number of issues released annually ( Supplementary Table S1 [available in the online version only]). Subscription costs ranged from $44.90 ( *Advances in Skin and Wound Care* ) to $1,518.00 ( *Plastic and Reconstructive Surgery* ). While a majority of journals had nominal to no submission/processing fees, OA APC publication ranged from $700.00 ( *Archives of Plastic Surgery* ) to $5,334.00 ( *Aesthetic Surgery Journal* ).

### Cost of Board Certification

The average total cost of becoming a board-certified PRS surgeon in the United States was estimated to be $9,045 ( Table 4 ). This includes USMLE fees ($2,955.00), ABPS board preparation ($250.00), ABPS certification ($1,030.00), ABPS written examination ($1,350.00), ABPS oral examination ($1,980.00), and estimated travel costs associated with the oral examination ($1,480). Annual ABPS recertification costs $410.00.

**Table 4:** Costs of becoming a board-certified plastic surgeon in the United States

Requirement	Cost ($)	Stage of training
**Board Examinations**		
USMLE Step 1	1,020	Medical School
USMLE Step 2	1,000	Medical School
USMLE Step 3	935	PGY-1 or PGY-2
**Subtotal**	**2,955**	**N/A**
**ABPS Board Preparation (Residency Examinations)**		
ASPS In-Service Exam for Residents (mandatory)	250	In Training (Resident)
Board Preparation Materials a	Variable	In Training (Resident)
**ABPS** b **Certification General Fees**		
Resident Registration/Evaluation of Training Fee	185	In Training (Senior Resident)
Application Registration Fee	450	In Training (Senior Resident)
Administrative Fee	250	In Training (Senior Resident)
Certificate Fee	145	In Practice
**Subtotal**	**1,030**	**N/A**
**ABPS Written Examination (Part 1)**		
Written Examination Fee	1,000	In Training (Senior Resident)
Written Examination Score Validation Fee	350	In Training (Senior Resident)
**Subtotal**	**1,350**	**N/A**
**ABPS Oral Examination (Part 2)**		
Oral Examination Case List Review Fee	685	First Year In Practice c
Oral Examination Fee	1,295	First Year In Practice c
Travel and Lodging d	1,480	First Year In Practice c
**Subtotal**	**3,460**	**N/A**
**ABPS Annual Dues** (excluded from “Total Cost” estimate)		
Continuous Certification Annual Fee e	410	Each year in practice
**Total cost** f	**$9,045**	

Abbreviation: USMLE, United States Medical Licensing Exam.

All costs are reported in $USD.

This figure is only representative of plastic surgeons who attended medical school in the United States.

a There are several other board preparation materials that prove costly, but are not listed here for brevity, and since they are not mandatory/fixed costs

b American Board of Plastic Surgery (ABPS).

c ABPS candidates are eligible for the Oral Examination after establishing practice and submitting a 9-month case list.

d All ABPS candidates are required to travel to Phoenix, AZ, for the Oral Examination. About $1,480 was the estimated travel expenses (airfare, hotel stay, transportation, and food) for a 2-night/3-day trip. This cost estimate was derived from a study by Williams et al. 19

e This fee is to be paid by board-certified plastic surgeons annually to maintain their certification by ABPS. For the purposes of this study, a one-time $410 cost was included in the “Total Cost” amount.

f It is important to recognize that these are the minimum fixed costs necessary to become a board-certified plastic surgeon. The annual cost of recertification ($410) was excluded from this “Total Cost” amount. The costs of medical school are excluded from this analysis. The costs of residency applications, medical school-away rotations, and other expenses associated with research and activities that are necessary to match into a plastic surgery residency are also included. It is estimated that the true costs amount to over $10,000 (excluding medical school tuition).

### Continuing Medical Education Costs

CME credits were recorded for PRS meetings and other online educational materials ( Supplementary Table S2 [available in the online version only]). The most expensive cost-per-CME credit activity was the VASPS annual meeting ($185.71 per 1 CME credit), while the cheapest was the American Society of Breast Surgeons' (ASBrS) annual meeting ($10.64 per 1 CME credit). Non-traditional methods of obtaining CME credit were significantly higher than traditional avenues, as most online ASPS resources yielded a cost-per-CME credit ratio of $200.00 per 1 CME credit, with the “ASPS Education Network: Quick Hits!” activity being the most expensive at $600.00 per 1 CME credit.

### Plastic and Reconstructive Surgery Costs Relative to Other Specialties

**Fig. 3:**
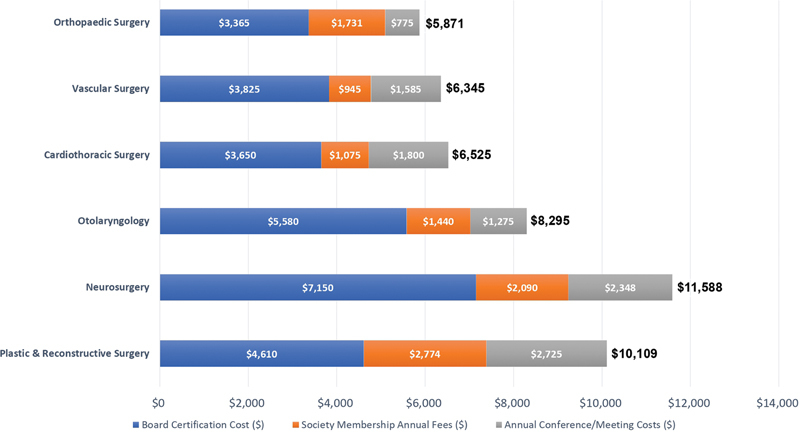
This figure represents the baseline academic engagement (BAE) costs of various surgical specialties. PRS ranked second highest among six key specialties, only behind neurosurgery. BAE was defined as the one-time cost of board certification, annual costs of two society memberships, and annual costs of two society conferences/meetings. Note that all costs are represented in United States dollars ($USD). PRS, plastic and reconstructive surgery.

When analyzing the costs of BAE across six surgical specialties, PRS ranked as the second most expensive specialty, behind only Neurosurgery. BAE costs demonstrated a clear tiered pattern, with Neurosurgery ($11,588) and PRS ($10,109) representing the highest-cost specialties, followed by Otolaryngology ($8,295) at an intermediate level, and Cardiothoracic ($6,525), Vascular ($6,345), and Orthopaedic Surgery ($5,871) representing the lowest relative cost burdens. Detailed cost breakdowns and a visual representation of the rankings are provided in Supplementary Table S3 (available in the online version only) and Fig. 3 , respectively.

## Discussion

### The Hidden Costs of Academic Engagement

Meaningful participation in academic PRS in the United States requires substantial personal financial investment. Costs, such as OA APCs, which can exceed $3,000 per article, and conference registration fees, ranging up to $1,000, are not reimbursed for medical trainees, junior faculty, and surgeons at non-academic hospitals. These costs add to the financial strain faced by medical trainees, including student loans and other research/extracurricular expenses, which have become somewhat necessary due to the rising competition to enter the field of PRS. For practicing surgeons, in many cases, these costs are almost a requirement in order to advance within the field.

### Society, Association, and Organizations Costs

With the rapid proliferation of knowledge within the field of PRS, conference attendance is critical to remain up to date on the latest innovations within the field. 17 21 22 Additionally, PRS has diversified into multiple subspecialty fields, including craniofacial surgery, aesthetic surgery, reconstruction/wound healing, advanced microsurgery, and gender affirming surgery, which has led many physicians to pursue advanced fellowship training to gain expertise within one specific area. 23 As a result, numerous subspecialty organizations have emerged, fostering more focused research efforts, the development of specialized practice guidelines, and highly specialized innovations ( Tables 1 and 2 ). Therefore, participation in niche conferences is imperative for surgeons to share knowledge, collaborate with peers, and advance patient care. It goes without saying that larger, more generalized meetings, such as Plastic Surgery The Meeting (PSTM) and the AAPS Annual Meeting, also remain valuable for delivering broad specialty-wide updates and fostering a global sense of community. 2 24 25 However, several plastic surgery societies, both large and small, are now struggling to maintain membership levels, as increasing financial burden and time constraints discourage active participation. With declining reimbursements, time constraints, and inflation, many surgeons may view it more financially and logistically practical to opt out of pricy memberships and conferences.

Ramanadham and Rohrich, and Carbullido et al emphasize that the value of conference attendance for early-career surgeons lies in building relationships with mentors that can blossom into lifelong friendships that are grounded in trust and respect. 17 Gaining exposure to new surgical techniques and skills can also help with board examination preparation and practice development. 17 27 If high costs continue to be a significant barrier for young surgeons to pursue these critical learning opportunities, the quality of care for patients is at risk of declining.

While aiming to better understand the rising costs of academic engagement is a major purpose of this study, it is also important to recognize the significant increase in the number of PRS organizations. Academic engagement in conferences leads to improved success with respect to career progression. However, many individuals from underresourced and underrepresented backgrounds may not have the financial means to engage in organized PRS in the United States, preventing their progression within the field. This may lead to diminished diversity, reduced access to evolving techniques, and missed opportunities for interdisciplinary collaboration. Data suggest that financial barriers are closely linked with socioeconomic and racial disparities in PRS. A study surveying applications to the 2022 integrated plastic surgery match demonstrated that applicants from households earning ≤$100,000 annually had significantly lower odds of receiving interview offers and matching into plastic surgery compared to their higher-income peers, while applicants from households earning ≥$300,000 comprised the largest proportion of respondents. 28 Another analysis of socioeconomic diversity among PRS trainees further revealed that fewer than 10% report childhood household incomes below $40,000. 29 These data reiterate well-documented disparities within the specialty and suggest that the cumulative costs of academic engagement may contribute to structural inequities across the PRS pipeline. 30 31 This study found that there is a lack of minority group societies, and thus there is a need for more organizations that are focused on helping support specific disadvantaged individuals, both within PRS and those aspiring to become plastic surgeons.

Internationally, support models for academic engagement differ from those in the United States. In some European health care systems, professional society participation and CME are often integrated into institution- or employer-supported frameworks, while several Asian societies employ a tiered membership structure or bundled educational programming to reduce financial burden on surgeons. For example, European Association of Plastic Surgeons/British Association of Plastic Reconstructive and Aesthetic Surgeons meetings often bundle CME and society membership, thereby mitigating the added costs for surgeons to engage in CME and conferences. Similar to the United States, Ayoade et al found that in low- to middle-income countries (LMICs), limited access to PRS journals, research skill development, and mentorship disproportionately negatively affect early-career surgeons. 32

### Journal Subscription and Submission Fees

PRS academic journals are responsible for disseminating new research, ideas, and perspectives from surgeons in the United States and globally. 33 34 Dr. Rodney J. Rohrich, a renowned leader in PRS, stated that “Plastic surgery is a specialty built on problem-solving and innovation, values starkly in-line with evidence-based medicine.” A core tenet of academic engagement in PRS involves conducting meaningful research that helps define new clinical and surgical practices. Publication of this work in reputable, high-impact journals is a pivotal way to share these innovations and perspectives. 35

However, the cost of publishing manuscripts has become increasingly expensive, especially if authors wish to publish OA, which is accompanied by an APC. While many academic plastic surgeons affiliated with large academic research institutions are reimbursed for publication expenses, those employed in smaller hospitals, community clinics, and private practices are disproportionately affected by these expensive research costs ( Fig. 2 ). High costs may deter those without dedicated research funding from sharing innovations that could benefit the broader PRS community. As both academic- and private practice-generated research is valuable, surgeons in all practice settings should have the ability to publish their work.

OA journals in PRS, such as in *PRS Global Open (PRS GO)* or *ASJ Open Forum* , have been highly beneficial to the PRS community, although they only comprise 18% of all PRS journals. The benefit is twofold: First, all individuals can access high-impact research studies without a journal subscription or single-article fee, and, second, individuals who have research studies rejected by non-OA journals still have an avenue through which to publish their meaningful work. 36 37 However, Yesantharao et al found that OA PRS journals were more likely to publish review articles and less likely to publish on subspecialty topics. 37 Additionally, Gowda et al, among many others, expressed concern over the quality of content of fully OA medical journals. 38 Therefore, it is essential that plastic surgeons and trainees have access to both subscription-based and OA articles to fully engage with new literature to advance their research, clinical decision-making, and professional development.

Subscription-based journals, which usually have annual subscriptions ranging from $500 to $1,500, can prove costly for trainees and younger surgeons who may rely on existing literature to learn approaches to a procedure, novel techniques, interesting presentations of cases, and how to navigate surgical/medical care for those patients. There is a definitive need for more financially feasible avenues to access published research.

### Baseline Costs of Board Certification and Continuing Medical Education

There are several fixed costs that plastic surgeons in the United States are required to pay to become board-certified and maintain certification. Recently, there has been an outcry among physicians regarding the extremely high costs of board certification. 8 39 With the total cost of PRS board-certification amounting to over $7,000, students and trainees are entering practice with increasingly large amounts of debt. In addition to loan repayment, young surgeons and those without institutional support must find ways to complete CME credit requirements and pay annual board renewal fees. The ABPS mandates that practicing plastic surgeons complete 125 Category 1 credits of CME over a 5-year period to maintain certification, which averages to about 25 CME credits per year. This study's data show that conferences offer a more feasible cost-per-CME credit ratio, giving surgeons more CME credit with less money spent. For surgeons who cannot afford to attend in-person conferences, online education modalities have a cost per CME credit ratio four times that of conferences/meetings. Once again, surgeons without institutional support are disproportionately affected as they “pay out of pocket” for CME, while academic surgeons can get reimbursed for attending PRS-related events that count towards CME requirements ( Fig. 2 ). 40

Although travel- and lodging-related expenses were not included in our cost calculations, these costs represent a substantial additional financial burden associated with academic participation. A recent study performed by Williams et al estimated total meeting attending costs using a standardized model incorporating hotel lodging, meals/incidental expenses, and travel-related costs. 19 Using this framework, average per-meeting travel and lodging expenses for a 2-night/3-day trip are estimated to be approximately $1,480 ($300 nightly hotel rate × 2 nights + $180 meals/incidentals + $580 flight + $120 parking), which excludes registration and membership fees. These costs may substantially increase total expenditures beyond those reported in this study, further amplifying the financial barriers faced by surgeons without institutional support or who are from geographically isolated regions.

Despite these financial challenges, board certification, engagement in professional societies, and participation in CME activities are all critical to a surgeon's development and play an integral role in ensuring that PRS surgeons are up-to-date and fully competent to deliver high-quality patient care. 40 Therefore, there must be a reform to lower the board certification fees and CME costs, which can pose significant barriers to certain populations of plastic surgeons. These financial pressures are further exacerbated by declining clinical reimbursements for academic surgeons, making it increasingly difficult to justify the out-of-pocket costs associated with maintaining academic involvement. Potential strategies to mitigate CME-related financial burden include bundled CME packages offered through professional societies, institutional or health system-subsidized CME access, and expanded use of society-based educational platforms that provide credits at a reduced, marginal cost. Such models may help preserve educational rigor while improving affordability, particularly for surgeons without formal academic support.

Many early-career surgeons are swayed towards choosing academic practice due to the extensive support in place for them to access academic events, conduct research, build their practice, and establish their reputation. Entering directly into community or private practice is currently a riskier option in terms of the financial hurdles and diminished support. This trend has been seen on a larger scale across several specialties in the past 10 to 15 years, with more practice consolidation and private equity presence in the market space. 41 42 However, compared to other specialties, private or community PRS practices remain relatively successful and play just as important a role in delivering patient care as their academic colleagues. Therefore, support for these plastic surgeons who do not receive institutional support is imperative in PRS, specifically. Additionally, the financial burden of academic engagement varies considerably by practice setting and career stage. Surgeons practicing in rural or geographically isolated regions may face higher overall costs due to greater travel distance, limited access to discounted regional society memberships or events, and increased time away from clinical practice when attending national meetings. Conversely, costs likely decrease as academic surgeons become more senior and gain higher academic rank since invited speaker roles, leadership positions within professional societies, or institutional recognition may result in waived registration fees, covered travel expenses, or subsidized participation. These variations highlight that individual financial impact from one surgeon to another can be shaped by several factors, including institutional affiliation, geographical location, and academic rank.

### Actionable Interventions

Targeted, evidence-based interventions may help mitigate financial barriers and improve representation within PRS. While intervention-focused research remains limited, there is evidence that financial support initiatives, such as travel stipends, housing assistance, and fee offsets for visiting sub-internships, can help increase access for students from underrepresented backgrounds and lower socioeconomic backgrounds. 43 44 Also, institution- and foundation-sponsored programs have reported improved recruitment of trainees from more diverse backgrounds. 43 45 Structured mentorship and educational initiatives within PRS, as well as analogous programs in other surgical specialties, have demonstrated gains in participant engagement and pipeline development. Building on these models, scalable approaches such as society-based fee waivers, tiered membership and meeting pricing structures, and pooled institutional or philanthropic grant mechanisms may help further reduce financial barriers to academic engagement.

### Limitations

This study has several limitations. This analysis represents a cross-sectional snapshot of costs and data during the 2024–2025 cycle; therefore, fees may fluctuate over time in response to inflation, organizational policy changes, or other broader economic factors. With regard to Fig. 2 and the overall study, this analysis did not account for differences in compensation between academic and private practice/community-based surgeons. Because practice settings involve distinct financial structures and income trade-offs, differences in institutional support for academic activities should not be interpreted as differences in net financial burden. Additionally, cost data were obtained from publicly available sources and may not reflect negotiated rates, member discounts, and institutional subsidies. Travel expenses, accommodation costs, and lost clinical revenue from time taken off work were not accounted for. Application and interview-related costs were excluded due to high variability. A perceived value or return on investment assessment was not performed, given differences in practice setting and career goals. Lastly, this study focused on major societies and journals and did not include other newly emerging societies.

### Future Directions

While ASPS has begun creating small, identity- or interest-based groups, future efforts should prioritize expanding these lower-cost societies. They offer early-career surgeons affordable opportunities to connect with like-minded peers, fostering inclusion and long-term professional growth. Future research should examine how costs influence early-career decisions, including the choice between academic and private practice. A cost–benefit or return-on-investment analysis of academic engagement would also be valuable. Moreover, since this study primarily focused on U.S.-based professional societies/organizations, expanding comparative analyses to international professional societies as well may help identify different strategies to reduce financial barriers. Lastly, future studies could employ longitudinal cost-tracking frameworks with periodic reassessment to monitor trends in academic engagement costs over time, evaluate the impact of inflation and policy changes, and inform evidence-based strategies to improve affordability and access.

## Conclusion

This review highlights the substantial and often underappreciated financial burden associated with academic engagement in PRS in the United States. From board certification and CME requirements to society memberships, conferences, and journal fees, these costs can disproportionately affect trainees, early-career surgeons, and those in non-academic settings. Without structural reforms or support systems, the current model risks excluding diverse voices and stifling innovation in the field. Greater transparency, cost-reduction efforts, and targeted support for underresourced surgeons are essential to ensure that academic PRS remains equitable, inclusive, and sustainable.
