# Supplementary material for: Costs of Academic Engagement in Organized Plastic and Reconstructive Surgery in the United States
Source: Arch Plast Surg. 2026 May 29;53(3):287–98. doi: 10.1055/a-2832-6764 (PMC13287933; doi:10.1055/a-2832-6764)
Supplement: Supplementary file 1 — Supplementary Material [file 10-1055-a-2832-6764-s25sep0137rev.pdf]

Thieme

Supplementary Table 1. Costs of Subscriptions and Submission to Major Plastic Surgery Journals

	Cost ($)				Journal Information			
**Journal Name:**	**Individual Online Subscription (per year)**	**Individual Print Subscription (per year)***	**Submission Fee**	**Open Access Fee/Article Processing Fee (APC)**	**Impact Factor**	**Year Journal Founded**	**Journal Type**	**Affiliation w/ Society**
**General PRS**								
Plastic & Reconstructive Surgery (PRS)	1100	1518	0	$3000/3800*****	3.2	1946	Hybrid Open Access******	American Society of Plastic Surgeons (ASPS)
*PRS Global Open***	OA**	OA**	N/A	$2,195	1.5	2013	Fully Open Access	Independent
Annals of Plastic Surgery	977	1108	100	$3750/4500*****	1.4	1978	Hybrid Open Access	Independent
Journal of Plastic, Reconstructive, and Aesthetic Surgery (JPRAS)	N/A	773	0	$3470/4500*****	2	1946	Hybrid Open Access	British Association of Plastic, Reconstructive, and Aesthetic Surgeons (BAPRAS)
*Archives of Plastic Surgery***	OA**	OA**	0	700	1.3	2012	Fully Open Access	Korean Society of Plastic and Reconstructive Surgeons & Affiliated Societies

Clinics in Plastic Surgery	N/A	565	0	N/A	1.8	1974	Subscription-Based Only	Independent
Seminars in Plastic Surgery	363	454	0	3450	2.3	1987	Hybrid Open Access	Independent
**Facial Plastic Surgery**								
Facial Plastic Surgery Clinics	387	441	0	N/A	1.9	1991	Subscription-Based Only	Independent
Facial Plastic Surgery	434	542	0	3450	1.1	1983	Hybrid Open Access	Independent
Facial Plastic Surgery & Aesthetic Medicine	287	311	0	3600	1.6	1999****	Hybrid Open Access	American Association of Facial Plastic and Reconstructive Surgeons (AAFPRS)
**Aesthetic Surgery**								
Aesthetic Surgery Journal (ASJ)	473	270	0	5334	3.1	1996	Hybrid Open Access	The Aesthetic Society
*Aesthetic Surgery Journal Open Forum (ASJOF)***	OA**	OA**	0	3051	1.6	2019	Fully Open Access	The Aesthetic Society

*Aesthetic Plastic Surgery******	419	N/A	0	4790	2	1976	Hybrid Open Access	ISAPS
**Breast Surgery**								
The Breast	OA**	OA**	0	3200	3.5	1992	Full Open Access	Australasian Society for Breast Disease (ASBD) & European Society of Breast Cancer Specialists (EUSOMA)
European Journal of Surgical Oncology (EJSO)	N/A	500	0	3810	3.5	1975	Hybrid Open Access	European Society of Surgical Oncology (ESSO)
**Hand Surgery**								
Journal of Hand Surgery (American Volume)	N/A	657	0	3450	1.9	1976	Hybrid Open Access	American Society for Surgery of the Hand (ASSH)
Journal of Hand Surgery Global Online	OA	OA	0	2500	2.1	2008	Fully Open Access	American Society for Surgery of the Hand (ASSH)
HAND	354	417	0	2500	1.8	2006	Hybrid Open Access	American Association for Hand

								Surgery (AAHS)
**Limb/Extremity/Reconstructive Surgery & Advanced Wound/Burn**								
Journal of Burn Care & Research	963	524	0	3968	1.5	1980	Hybrid Open Access	American Burn Association (ABA)
Advances in Skin and Wound Care	39.95	44.9	0	3000	1.7	1988	Hybrid Open Access	American Professional Wound Care Association (APWCA)
Wound Repair and Regeneration	362	N/A	0	3750	3.8	1993	Hybrid Open Access	Wound Healing Society (WHS)
**Microsurgery**								
Journal of Reconstructive Microsurgery	N/A	617	0	3450	2.2	1984	Hybrid Open Access	Independent
*Journal of Reconstructive Microsurgery Open***	OA**	OA**	0	3050	None Assigned	2016	Fully Open Access	Independent
Microsurgery	273	N/A		3300/3950*****	1.5	1980	Hybrid Open Access	Independent

**Craniofacial**								
Journal of Craniofacial Surgery	1978	2002	100	3330/3700*****	1	1990	Hybrid Open Access	Independent
Journal of Cranio-Maxillofacial Surgery	0	426	0	3784	2.1	1987	Hybrid Open Access	European Association for Cranio-Maxillofacial Surgery
The Cleft Palate-Craniofacial Journal	N/A	410	0	4150	1.2	1964	Hybrid Open Access	American Cleft Palate-Craniofacial Journal

Note: OA = Open Access

*Print subscriptions oftentimes include online access as well

**Journal is only Open Access

***Free access for ISAPS Members

****JAMA Facial Plastic Surgery was founded in 1999, but then merged to form Facial Plastic Surgery and Aesthetic Medicine

*****$3000 CC BY-NC-ND license & $3,800 CC BY license

******Hybrid Open Access means the authors have the option to choose subscription only or open access. Therefore, the open access/APC fee listed is only to be paid if the authors select the Open Access publication option.

Thieme

Supplementary Table 2. CME Credit Sources and Cost-per-CME Credit Values

		Cost ($)		
**Type**	**CME Source**	**Cost (member/nonmember if applicable)**	**CME Credits***	**Cost-per-CME Credit (member/nonmember if applicable)**
CME Acquired via Meetings	**National**			
	ASPS Annual Meeting - Plastic Surgery the Meeting (PSTM)**	1450/2115	75	19.33/28.20
	AAPS Annual Meeting	1050/1250	29.25	35.89/42.73
	PSRC Annual Meeting	800/825	15.75	50.79/52.38
	**Regional/State**			
	CSPS Annual Meeting	600/1200	20.25	26.93/59.26
	SESPRS Annual Scientific Meeting	1180/1780	21	56.19/84.76
	NYRSPS Annual Meeting	0/50	6	0/8.33
	FSPS Annual Meeting	1495/1700	18.75	79.73/90.66
	TSPS Annual Meeting	750/825	12.25	61.22/67.35
	VASPS Annual Meeting	975/1175	5.25	185.71/223.81
	NWSPS Annual Meeting	600/900	20.75	28.92/43.37
	NESPS Annual Meeting	650/750	19.25	33.77/38.96
	NESPRS Annual Meeting	825/925	9.75	84.62/94.87
	OVSPS Annual Meeting	700/700	14	50/50

	RHIS Annual Meeting	300/500	7.5	40/66.67
	MSPS Annual Meeting	0/0	None	N/A
	MWSPS Annual Meeting	600/800	10.5	57.14/76.19
	**Aesthetic Surgery**			
	The Aesthetic Society Annual Meeting - The Aesthetic Meeting	1275/1875	33.25	38.35/56.39
	ISAPS Annual Meeting	650/1000	36.5	17.81/27.40
	AACS Annual Meeting	1399/1699	None	N/A
	Baker Gordon Annual Educational Symposium (The Aesthetic Society, ISAPS, and ASPS)	1800/1850	20	90/92.5
	**Breast Surgery**			
	ASBrS Annual Meeting	795/1395	74.75	10.64/18.66
	**Hand Surgery**			
	ASSH Annual Meeting	1045/1255	33.75	30.96/37.19
	AAHS Annual Meeting	925/1325	24.5	37.76/54.08
	**Limb/Extremity/Reconstructive Surgery**			
	Diabetic Limb Salvage (DLS) Annual Meeting	799/899	20.75	38.51/43.33
	**Advanced Wound/Burn**			
	WHS Annual Meeting	639/639	23.75	26.91/26.91

	ABA Annual Meeting	999/1099	26.5	37.40/41.47
	**Microsurgery**			
	ASRM Annual Meeting	950/1400	31	30.65/45.16
	ASPN Annual Meeting		15.75	
	**Craniofacial**			
	ASCFS/ACPA Combined Annual Meeting	650/965	27.75	23.42/34.77
	ASMS Annual Meeting	**	**	**
	ISCFS Annual Meeting	1300/1250	25	52/50
	Migraine Surgery Symposium	**	**	**
	**Gender Affirming Surgery**			
	WPATH Annual Meeting	755/920	14.5	52.07/63.45
	**Identity Groups**			
	Woman Plastic Surgery Symposium	800/800	13.25	60.38/60.38
CME Acquired Online***	ASPS Education Network Hubs: Online Education Modules	200	1	200
	ASPS Education Network: Journal CME Articles	200	1	200
	ASPS Education Network: Quick Hits!	150	0.25	600

Thieme

CME Acquired via Assessments	ASPS In-Service/Self-Assessment Surgeons Exam	555/655	37.5	14.80/17.46
	ASPS L3E Aesthetic In-Service Exam	495/595	30	16.50/19.83

*CME credits for each meeting noted are the maximum amount of CME that could possible be earned by attending the meeting and all qualifying events/sessions

**The American Society of Maxillofacial Surgeons (ASMS) & Migraine Surgery Symposium annual meeting takes place during ASPS's PSTM, and their CME credit is a part of the 75 total CME credits received for attending the PSTM conference

***Several societies and organizations offer "enduring" CME credit, which is educational content that is available over time (in the form of online/recorded formats) which counts for CME credit/hours. For brevity, all online sources of CME credit are not listed here, but rather only major ones from the leading ASPS society.

Supplementary Table 3. Baseline Academic Engagement (BAE) Costs Among Various Surgical Specialties

		**Specialty**					
		**Plastic & Reconstructive Surgery**	**Neurosurgery**	**Otolaryngology**	**Cardiothoracic Surgery**	**Vascular Surgery**	**Orthopaedic Surgery**
**Baseline Academic Engagement (BAE) Components**	**Board Certification Cost (One-Time Fee)**	**ABPS Board Preparation/ Application/ Certification Fees – $1,280,** Written Exam - 1350, Oral Exam - 1980 (Please see Table 4)	Application Fee - 2000, Oral Examination - 4000, Written Exam - 1000, Neuroanatomy Exam - 150	Application & Exam Fee - 3580, Oral Exam Fee - 2000	Application Fee - 650, Written Exam Fee - 1500, Oral Exam Fee - 1500	Application Fee - 550, Qualifying Exam - 1575, Certifying Exam - 1700	Part I - 1040, Part II - 975+1350

	**Society Membership #1 Annual Fee**	ASPS Annual Dues - 1299	AANS Annual Dues - 1,100	AAOHNS Annual Dues - 1090	STS Annual Dues - 750	SVS Annual Dues - 760	AAOS Annual Dues - 1131
	**Society Membership #2 Annual Fee**	TAS Annual Dues - 1475	CNS Annual Dues - 990	ARS Annual Dues - 350	AATS Annual Dues - 325	SCVS Annual Dues - 185	AAHKS Annual Dues - 600
	**Annual Conference/Meeting #1 Cost**	ASPS PSTM Annual Meeting - 1450	AANS Annual Meeting - 1,149	AAOHNS Annual Meeting - 775	STS Annual Meeting - 900	SVS Annual Meeting - 925	AAOS Annual Meeting - 275
	**Annual Conference/Meeting #2 Cost**	TAS Annual Meeting - 1275	CNS Annual Meeting - 1,199	ARS Annual Meeting - 500	AATS Annual Meeting - 900	SCVS Annual Meeting - 650	AAHKS Annual Meeting - 500
	**Total Cost of BAE:**	10,109	11,588	8,295	6,525	6,345	5,871

Abbreviations: American Society of Plastic Surgeons (ASPS), The Aesthetic Society (TAS), American Association of Neurological Surgeons (AANS), Congress of Neurological Surgeons (CNS), American Association of Otolaryngology & Head and Neck Surgery (AAOHNS), American Rhinologic Society (ARS), Society of Thoracic Surgeons (STS), American Association of Thoracic Surgeons (AATS), Society for Vascular Surgery (SVS), Society for Clinical Vascular Surgery (SCVS), American Academy of Orthopaedic Surgeons (AAOS), and American Association for Hip and Knee Surgeons (AAHKS)

Note: This data was used to generate **Figure 3.** All costs are represented in United States Dollars ($ USD)
